# CircRNA-Associated CeRNAs Regulatory Axes in Retinoblastoma: A Systematic Scoping Review

**DOI:** 10.3389/fonc.2022.910470

**Published:** 2022-07-05

**Authors:** Mohammad Reza Asadi, Marziyeh Sadat Moslehian, Hani Sabaie, Mirmohsen Sharifi-Bonab, Parvin Hakimi, Bashdar Mahmud Hussen, Mohammad Taheri, Azadeh Rakhshan, Maryam Rezazadeh

**Affiliations:** ^1^ Clinical Research Development Unit of Tabriz Valiasr Hospital, Tabriz University of Medical Sciences, Tabriz, Iran; ^2^ Department of Medical Genetics, Faculty of Medicine, Tabriz University of Medical Sciences, Tabriz, Iran; ^3^ Woman’s Reproductive Health Research Center, Tabriz University of medical sciences, Tabriz, Iran; ^4^ Department of Pharmacognosy, College of Pharmacy, Hawler Medical University, Erbil, Iraq; ^5^ Center of Research and Strategic Studies, Lebanese French University, Erbil, Iraq; ^6^ Urology and Nephrology Research Center, Shahid Beheshti University of Medical Sciences, Tehran, Iran; ^7^ Institute of Human Genetics, Jena University Hospital, Jena, Germany; ^8^ Cancer Research Center, Shahid Beheshti University of Medical Sciences, Tehran, Iran

**Keywords:** retinoblastoma, circular RNA, CeRNA, sponge, circ_0000527, circ_0000034, circTET1

## Abstract

Retinoblastoma (RB) is one of the most common childhood cancers caused by RB gene mutations (tumor suppressor gene in various patients). A better understanding of molecular pathways and the development of new diagnostic approaches may lead to better treatment for RB patients. The number of studies on ceRNA axes is increasing, emphasizing the significance of these axes in RB. Circular RNAs (circRNAs) play a vital role in competing endogenous RNA (ceRNA) regulatory axes by sponging microRNAs and regulating gene expression. Because of the broadness of ceRNA interaction networks, they may assist in investigating treatment targets in RB. This study conducted a systematic scoping review to evaluate verified loops of ceRNA in RB, focusing on the ceRNA axis and its relationship to circRNAs. This scoping review was carried out using a six-step strategy and the Prisma guideline, and it involved systematically searching the publications of seven databases. Out of 363 records, sixteen articles were entirely consistent with the defined inclusion criteria and were summarized in the relevant table. The majority of the studies focused on the circRNAs circ_0000527, circ_0000034, and circTET1, with approximately two-fifths of the studies focusing on a single circRNA. Understanding the many features of this regulatory structure may help elucidate RB’s unknown causative factors and provide novel molecular potential therapeutic targets and medical fields.

## 1 Introduction

Retinoblastoma (RB) is the most frequent type of childhood eye tumor. RB is caused by a mutation in the tumor suppressor gene Rb1. It is well-known that RB is a typical illustration of Knudson’s two-hit theory (1). It is a rapidly developing tumor, with a doubling period of 15 days in highly young patients (2). The global prevalence of RB ranges between 1/15,000 and 1/20,000 live births (3). The International Classification of RB categorizes intraocular tumors based on tumorigenesis and pattern. They are divided into five categories: A through E. The odds of saving the eye diminish as the group progresses from A to E. Enucleation is favored in unilateral patients (tumor in a single eye) when the malignancy reaches Groups D and E. In bilateral patients (tumors in both eyes), the eye with the most advanced tumor is enucleated (4). External beam radiation, episcleral plaque radiotherapy, enucleation, cryotherapy, and photocoagulation were formerly the standard therapies for children with RB (5). RB therapy has evolved significantly during the last decade. In this regard, with shifting views on radiotherapy concerns, intravenous and intra-arterial chemotherapies have become the cornerstone of RB treatment since they have been proven to efficiently reduce tumor size, metastasis, and preserve eyesight (6). However, their clinical applicability is restricted owing to the possibility of systemic toxicity, drug resistance, and fast blood clearance (7). Remarkably, attention to the molecular mechanisms like competing endogenous RNA (ceRNA) axes involved in all RB processes, from formation to even resistance to chemotherapy, provides the basis for this disease to be recognized as more fundamental.

CeRNA are transcripts that compete for shared microRNAs to cross-regulate each other (miRNAs) (8). The latter are short RNAs of 21–23 nucleotides in length that may lead Argonaute proteins to target transcripts by base pairing, causing them to degrade or repress translation (9, 10). Franco-Zorrilla et al. described a phenomenon known as ‘target mimicry’ in which a non-coding RNA in plants may sequester miR-399 and de-repress its target (11). Not long after, Ebert et al. discovered a similar phenomenon in animal cells. Ectopic production of a miRNA that has a large number of binding sites (also referred to as miRNA response elements (MREs)) results in miRNA sequestration that is hardly visible but leads to a 1.5- to 2.5-fold overexpression of the targets of the miRNA in this research (12). As a result, the term “RNA sponge” was created to characterize the phenomenon of miRNAs being absorbed by overexpressed MRE-containing transcripts. Following that, the RNA sponge phenomenon was found in various malignancies (13, 14). The term ‘ceRNA’ was created in 2011 to characterize this additional layer of posttranscriptional control (15).

Circular RNA (circRNA) is a non-coding RNA that primarily regulates biological processes *via* gene control. CircRNAs contain many binding sites for miRNA and hence operate like sponge, absorbing miRNAs (16). CircRNA may also interact with other RNAs through base pairing (17). Furthermore, by interacting with proteins, circRNA may limit their function (18). Although circRNA is a form of non-coding RNA, under specific circumstances, it may serve as a translation template for protein synthesis and yield functional proteins (19). CircRNAs have recently been discovered to affect host gene expression, interact with RNA-binding proteins that govern transcription, function in cis transcriptional regulation, and even regulate and control alternative splicing (20). The fact that ceRNA interaction networks are multifactorial means that they may be valuable in investigating complicated diseases such as RB in terms of therapeutic targets since by targeting just one of them, the levels of several disease-related RNAs change simultaneously (21). We performed a systematic scoping review in this study to look into validated ceRNA loops in RB. The focus of our study was the circRNA-ceRNA axes, which have been associated with RB etiology and might be utilized as therapeutic targets.

## 2 Methods

The approach for this study was based on Arksey and O’Malley’s (22) scoping review framework, which was subsequently revised by Levac et al. (23). This process consists of five unique steps: (1) identifying the research question, (2) identifying relevant studies, (3) study selection, (4) data charting, and (5) collating, summarizing, and reporting results. We did not use consultation in our research, an optional sixth stage in the scoping review. The Preferred Reporting Items for Systematic Reviews and Meta-Analyses Extension for Scoping Reviews (PRISMA-ScR) Checklist also effectively guided this review (24).

### 2.1 Identifying the Research Question

The purpose of this study was to compile and organize the existing information on circRNA-associated ceRNA loops in RB. In order to achieve this goal, we aimed to respond to the following question: What is known about circRNA-associated ceRNA regulatory axes in RB based on the extant literature?

### 2.2 Identifying Relevant Studies

An initial restricted search of PubMed and Embase was carried out, and then the keywords in the title and abstract, as well as the index terms that were used in the articles, were evaluated for significance. A second search was conducted throughout the PubMed, Embase, Scopus, Web of Science, and Cochrane databases without any restrictions, using keywords, MeSH, or Emtree terms identified in the original search. Additionally, searches were conducted in two gray-listed (i.e., difficult to find or unpublished) literature databases: Google Scholar and ProQuest. The most recent search was conducted on March 23, 2022. We also looked through the reference lists of relevant literature and reviewed papers for additional sources.

### 2.3 Study Selection

The included papers met the following criteria: (1) explicitly discussing the circRNA-associated ceRNA axis in RB, (2) published in English, and (3) be original research. The exclusion criteria were: (1) non-RB or any ocular tumor studies, (2) studies that did not utilize human specimens or cell lines, and (3) studies that did not use a molecular approach to verify the ceRNA loop components. The title and abstract of each article were separately reviewed for eligibility according to the criteria mentioned earlier by two reviewers (MRA, HS). The remaining publications’ entire texts were assessed, and those that met the eligibility requirements were included in the final data analysis. Any differences were resolved *via* conversation or, if necessary, with a third reviewer (MR).

### 2.4 Data Charting

Two reviewers (MRA and HS) extracted data separately into a predesigned charting template in Microsoft Excel. It listed the first author, the year of publication, the country, the type of study, the cell line(s), human samples, animal models, methods, ceRNAs, shared miRNAs (s), and the key findings.

### 2.5 Collating, Summarizing, and Reporting the Results

We conducted both quantitative and qualitative analyses. We produced a descriptive numerical overview of the features of the included articles for the quantitative section. As part of the qualitative analysis, we wrote a narrative review of the current information that answered our research question, focusing on how important the results were in a larger context, as suggested by Levac et al. (23).

## 3 Results

### 3.1 Search Results

**Figure 1:**
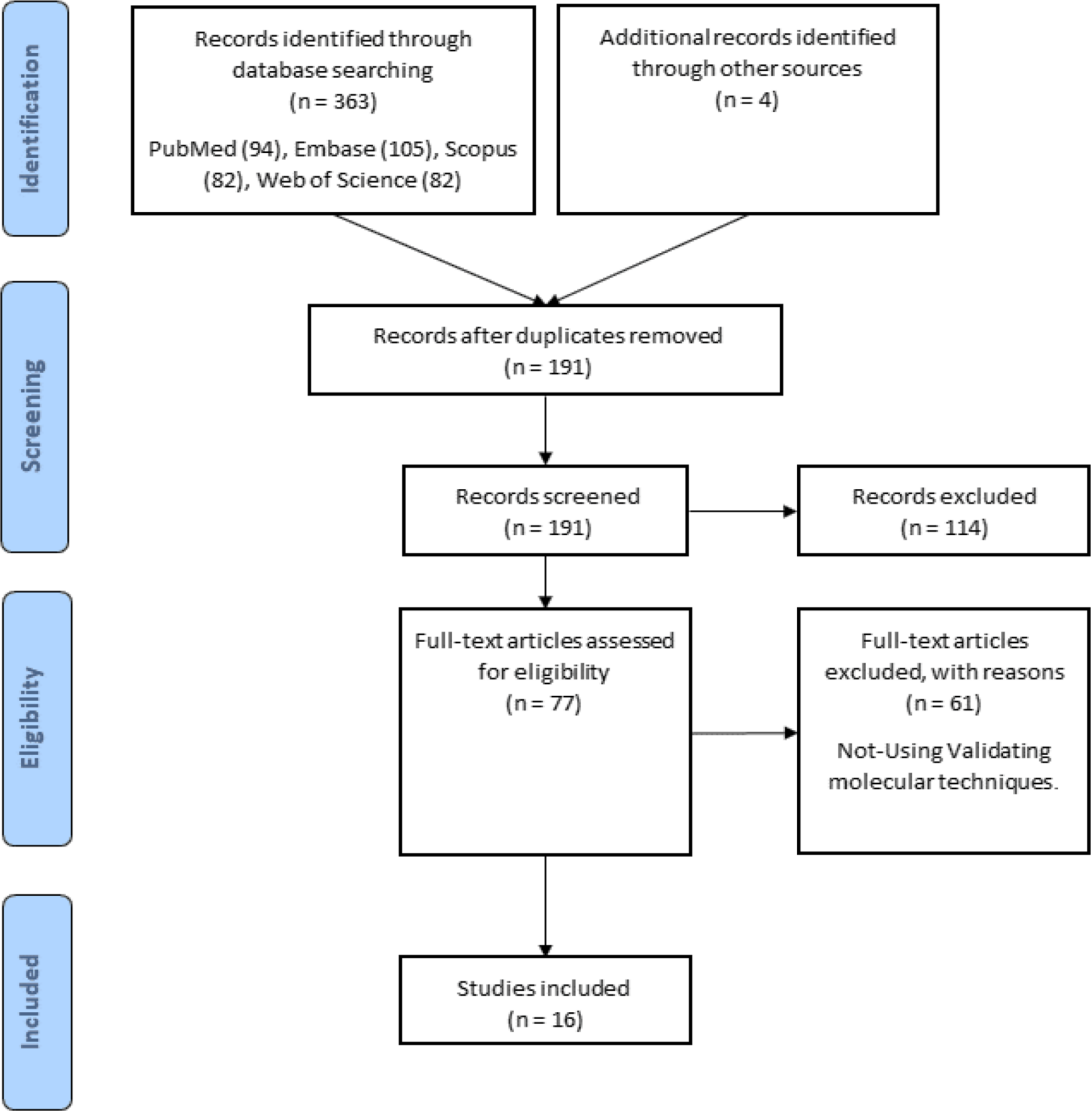
Search strategy flow chart based on the PRISMA flow diagram.

The flow chart in **Figure 1** depicts the various steps involved in locating eligible studies. A total of 363 articles were identified from multiple sources, of which 176 were duplicates. One hundred seventy-one articles were omitted due to their insignificance. After reading the full texts of the remaining 16 articles for the purpose of evaluation, the results are presented in **Table 1**.

**Table 1:** Circular RNAs in Retinoblastoma ceRNA axes.

First author	Year of publication	Country	Type of study	Human samples	Cell line(s)	Animal model	Methods	CeRNAs	SharedmiRNA(s)	Key findings	ref
Du et al.	2019	China	Human study, Cell culture, Animal study	60 RB tissues and 60 normal retina tissues	Y79 HXO-RB44 SO-Rb50 WERI-Rb-1 ARPE-19	athymic mice	Cell transfection, q-PCR, Western blot, Cell proliferation assays, Nuclear/cytoplasmic fractionation, Dual‐luciferase reporter assay	circ_ODC1 and SKP2	miR‐422a	It was discovered that SKP2 was disinhibited by circ_ODC1 from miR422a, which resulted in increased RB proliferation.	(25)
Chen et al.	2020	China	Human study, Cell culture	30 human RB samples and 30 adjacent normal samples	Y79 HXO-RB44 SO-Rb50 WERI-Rb-1	–	q-PCR, CCK-8 assay, TUNEL assay, Transwell assay, Dual-luciferase reporter gene assay, RIP assay, Western blot	circ_0000527 and BCL-2	miR-646	Circ_0000527 expression was considerably higher in RB samples compared to normal tissues, whereas miR-646 expression was significantly lower. Circ_0000527 increased the viability, migration, and invasion of RB cells and sponged miR-646 to regulate BCL-2 expression.	(26)
Liu et al.	2020	China	Human study, Cell culture, Animal study	32 RB tissues and 6 normal retina tissues	Y79 HXO-RB44 SO-Rb50 WERI-Rb-1 ARPE-19	SCID mice	q-PCR, RNase R Treatment, Cell Transfection, Cell Proliferation Assay, Cell Apoptosis Assay, Cell Migration and Invasion Assay, Western Blot, Dual-Luciferase Reporter Assay, RIP Assay	circ_0000034 and STX17	miR-361-3p	Circ_0000034 increased STX17 levels by sponging miR-361-3p. Circ_0000034 knockdown suppressed RB cell growth by influencing the miR-361-3p/STX17 axis.	(27)
Sun et al.	2020	China	Human study, Cell culture	38 RB tissues and 12 normal retina tissues	Y79 SO-Rb50 WERI-Rb-1 ARPE-19	–	q-PCR, Subcellular fractionation location, EdU assay, Wound healing assay, Transwell invasion assay, RIP assay, Luciferase reporter assay	circ_0000034 and miR-361-3p targets	miR-361-3p	Circ_0000034 was overexpressed in RB, and increased expression levels of Circ_0000034 are related to a malignant phenotype. Circ_0000034 appeared to function as a ceRNA in RB *via* miR-361-3p sponging.	(28)
Wang et al.	China	2020	Human study, Cell culture, Animal study	15 RB tissues and 10 normal retina tissues	Y79 WERI-Rb-1	BALB/c nude mice	q-PCR, Transfection Assay, Colony Formation Assay, Flow Cytometry Analysis, Transwell Assay, Western Blot, Dual−Luciferase Reporter Assay	CircDHDDS and WNT3A	miR−361−3p	CircDHDDS by sponging miR-361-3p regulate WNT3A expression. As a result, the circDHDDS/miR-361-3p/WNT3A axis promoted RB development by regulating RB cell growth, cell cycle program, migration, and invasion.	(29)
Zhang et al.	China	2020	Human study, Cell culture	45 human RB samples and 45 adjacent normal samples	Y79 SO-Rb50 RB355 WERI-Rb-1 ARPE-19	–	q-PCR, CCK−8 assay, Flow cytometry, Transwell migration and invasion assays, Western blot, RIP assay, Dual luciferase reporter assay	Circ_0000527 and LRP6	miR-646	Circ_0000527 overexpression promoted RB cell growth, migration, and invasion while suppressing cell apoptosis, whereas circ_0000527 knockdown suppressed malignant biological behavior. Circ_0000527 regulated LRP6 expression by directly targeting miR-646.	(30)
Fu et al.	China	2021	Human study, Cell culture, Animal study	30 RB tissues and 30 normal retina tissues	Y79 WERI-Rb-1 ARPE-19	BALB/c nude mice	q-PCR, Nuclear and cytoplasmic fraction assay, Cell viability assay, Colony formation assay, Flow cytometry, Scratch assay, Transwell assay, Western blot, Dual-luciferase reporter assay, RNA pull-down assay	CircTET1 and miR-492 and miR-494-3p targets	miR-492 miR-494-3p	CircTET1 expression was decreased in RB tissues and cells, whereas its overexpression inhibited RB cell progression. CircTET1 acted as a sponge for miR-492/miR-494-3p, which inhibited the Wnt/-catenin pathway.	(31)
Huang et al.	China	2021	Human study, Cell culture, Animal study	23 RB tissues and 16 normal retina tissues	Y79 SO-Rb50 WERI-Rb-1 ARPE-19	BALB/c nude mice	q-PCR, RNase R assay, Cell proliferation, Flow cytometry, Transwell assay, Wound healing assay, Dual-luciferase reporter assay, Western blot, IHC	Circ-E2F3 and ROCK1	miR-204-5p	Circ-E2F3 as a tumor promoter promoted RB proliferation, metastasis, and apoptosis by modulating the miR-204-5p/ROCK1 axis.	(32)
Jiang et al.	China	2021	Cell culture, Animal study	–	Y79 SO-Rb50 WERI-Rb-1 ARPE-19 SO-Rb70	BALB/c nude mice	q-PCR, Nuclear and cytoplasmic fraction assay, Colony formation assay, Flow cytometry, Transwell analysis, Caspase-3/caspase-9 activity assay, Dual-luciferase reporter assay, RIP assay, RNA pull-down assay, Western blot	circ_0099198 and LRP6	miR-1287	Circ_0099198 depletion suppressed RB cell progression by regulating the miR-1287/LRP6 axis. Circ_0099198 aided RB progression through sponging miR-1287 and increasing LRP6 expression.	(33)
Xu et al.	China	2021	Human study, Cell culture, Animal study	55 human RB samples and 55 adjacent normal samples	Y79 WERI-Rb-1 ARPE-19	BALB/c nude mice	q-PCR, Subcellular fraction assay, Cell transfection, CCK-8 assay, Colony formation assay, Transwell assay, Western blot, Dual-luciferase reporter assay, RIP assay	CircMKLN1 and PDCD4	miR-425-5p	CircMKLN1 expression was reduced in RB tissues and cells. Elevated levels of circMKLN1 were linked to a better outcome in RB patients. In addition, circMKLN1 is a target gene for miR-425-5p. Silencing PDCD4 may improve the inhibitory roles of circMKLN1 in RB cell development and metastasis.	(34)
Jiang et al.	China	2021	Human study, Cell culture, Animal study	30 human RB samples and 30 adjacent normal samples	Y79 SO-Rb50 WERI-Rb-1 ARPE-19	BALB/c nude mice	q-PCR, CCK−8 assay, Transwell assay, Flow cytometry assay, Caspase−3 activity assay, Western blot, Dual−luciferase reporter assay, RIP assay, RNA pull−down assay	circ_0000034 and ADAM19	miR−361−3p	Circ_0000034 Silencing suppressed RB progression by down-regulating ADAM19 through miR-361-3p sponging.	(35)
Zhang et al.	2021	China	Human study, Cell culture, Animal study	47 RB tissues and 9 normal retina tissues	Y79 WERI-Rb-1 HXO-Rb44 ARPE-19	nude BALB/c mice	RNase R or actinomycin D treatment, q-PCR, Cell transfection, CCK8 assay, EdU assay, Flow cytometry, Wound healing assay for cell migration analysis, Transwell invasion assay, Western blot assay, Dual-luciferase reporter assay, RIP assay	Circ_0075804 and PEG10	miR-138-5p	Circ_0075804 assisted the malignant characteristics of RB cells by binding to miR-138-5p and causing PEG10 expression.	(36)
Liang et al.	2021	China	Cell culture, Animal study	–	NR	Nude mice	q-PCR, Western blot assay, IHC, RNase R assay and actinomycin D assay, CCK-8 assay, colony formation assay, Wound healing assay and transwell assay, Flow cytometry analysis, Dual-luciferase reporter assay and RIP assay	Circ_0000527 and SMAD2	miR-1236-3p	Circ_0000527 targeted SMAD2 by sponging miR-1236-3p. The level of miR-1236-3p was reduced in RB tissues and cells. miR-1236-3p overexpression inhibited RB cell progression, with SMAD2 elevation abrogating the effect.	(37)
Yu et al.	2021	China	Human study, Cell culture, Animal study	27 RB tissues and 19 normal retina tissues	hTERT-RPE1 Y79 WERI-Rb-1 ARPE19	BALB/c nude mice	q-PCR, Cell proliferation assay, Colony formation assay, Flow cytometry, Transwell assay, Scratch assay, Dual-luciferase reporter assay, RIP assay, Western blot assay	Circ_0000527 and XIAP	miR-98-5p	Circ_0000527 expression was increased in RB tissues and cells, and its silencing inhibits RB cell development. *In vivo*, silencing Circ_0000527 impeded RB growth. It controlled RB progression *via* the miR-98-5p/XIAP axis.	(38)
Zheng et al.	2021	China	Human study, Cell culture, Animal study	33 RB tissues and 21 normal retina tissues	HRA Y79 WERI-Rb-1	BALB/c nude mice	q-PCR, RNase R treatment, Cell proliferation assay, Flow cytometry, Transwell assay, Dual-luciferase reporter assay, Western blot, IHC, TUNEL assay	circ-FAM158A and SLC7A5	miR-138–5p	In RB, circ-FAM158A and SLC7A5 were overexpressed, while miR-138-5p was down-regulated. Circ-FAM158A silencing inhibited RB cell growth, invasion, and migration while promoting G0/G1 cell cycle arrest and apoptosis. Circ-FAM158A acted as an oncogene in the RB by sponging miR-138-5p, which regulated SLC7A5 expression.	(39)
Zuo et al.	2022	China	Human study, Cell culture, Animal study	NR	NR	BALB/c nude mice	q-PCR, CCK-8 assay, colony formation assay, flow cytometry assay, dual-luciferase reporter assay, RIP assay, western blot	circ_0000527 and HDAC9	miR-27a-3p	Down-regulation of circ_0000527 inhibited the progression of RB by regulating the miR-27a-3p/HDAC9 pathway, which may be related to the inactivation of the PI3K/AKT pathway.	(40)

### 3.2 Study Characteristics

**Figure 2:**
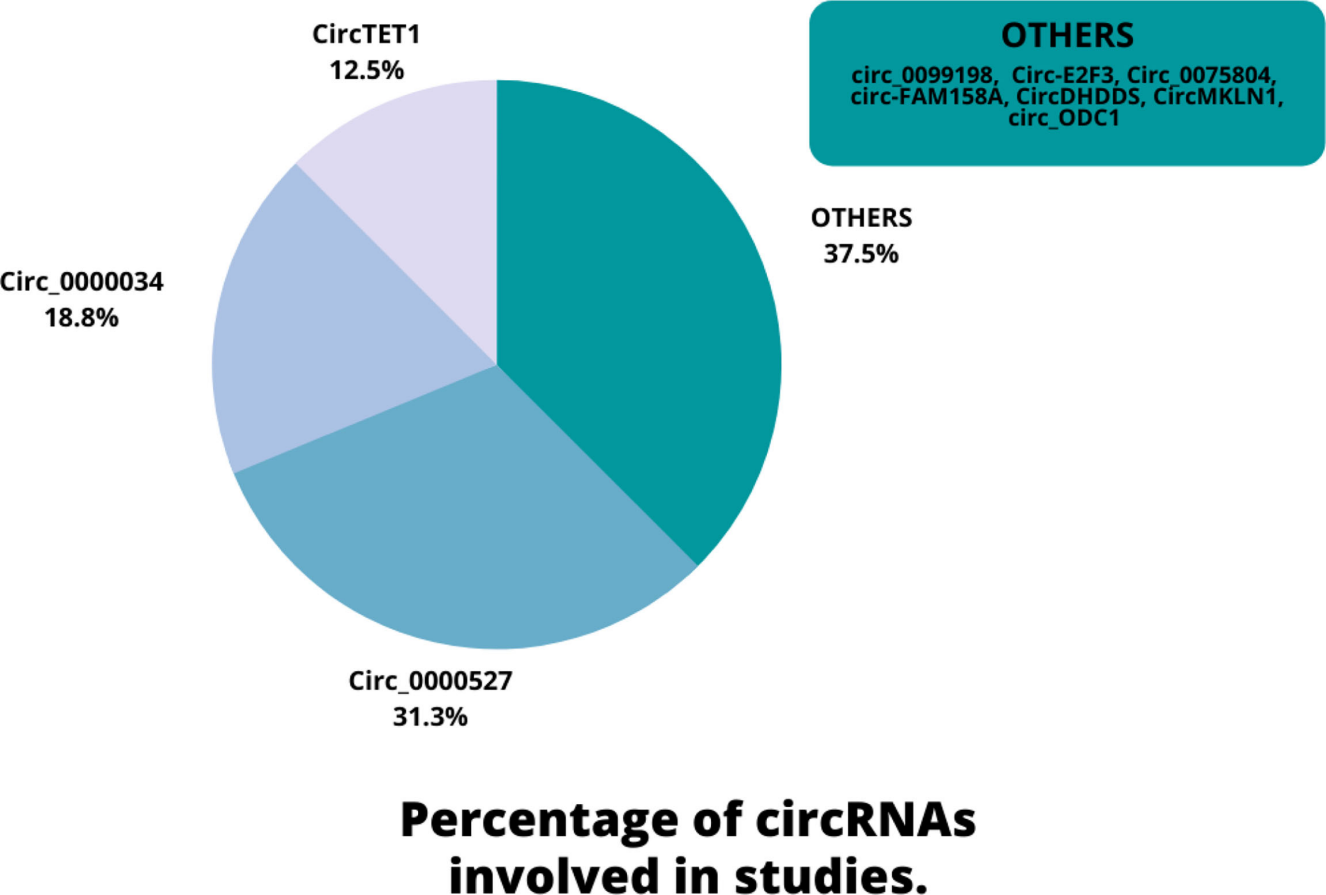
An overview of the circRNAs proportion is considered in the qualified studies.

Eligible studies were published between 2019 and 2022. These studies included 465 samples of RB patients and 343 healthy controls, based on the mentioned number. The gender of patients and controls is rarely mentioned. An animal model was used in 13 studies, and mice were the only animals involved. Weri-Rb-1, Y79, SO-RB50, SO-Rb70, RB355, HRA, HXO-RB44, ARPE-19, and hTERT-RPE1 are among the cell lines used in these studies. **Figure 2** depicts the frequency of circRNAs. Notably, the distribution of studies is restricted to only one country, namely China.

## 4 Discussion

### 4.1 CircRNA Features and Biogenesis

A recent study has shown that circRNAs are commonly formed from one to five exons, with lengths ranging from just a few hundred to thousands of nucleotides (41). CircRNAs produced *via* back-splicing have numerous fundamental properties: I. Circular RNAs have a closed ring shape, which means they lack either 5′–3′ polarity or a polyadenylated tail, making them resistant to exonuclease degradation and much more stable than linear RNA (42). II. CircRNAs are frequently expressed in eukaryotic cells, and high-throughput sequencing has shown that more than one million circRNAs exist in human tissues (43). III. The majority of circRNAs are found in the cytoplasm, with a minor number of circRNAs found in the nucleus as well (44). IV. The majority of circRNAs contain substantially conserved sequences across various species (45). V. CircRNAs demonstrate tissue-and developmental stage-specific and dynamic expression patterns (46). VI. CircRNAs act as transcriptional or posttranscriptional regulators (44).

It is critical to understand how circRNAs are created in order to classify them. In conjunction with the activation of the spliceosomal machinery, canonical splicing is a process in which the introns of precursor messenger RNAs (pre-mRNAs) are removed, and exons are then joined to generate mature messenger RNAs (47). In contrast to canonical splicing of mRNAs, circular RNAs are produced by nonsequential back-splicing of pre-mRNAs by spliceosomes rather than conventional splicing (5). Back-splicing is the process of reversibly joining the upstream 5′ splice donor site to the downstream 3′ splice acceptor site to form a covalently closed structure (5, 48). According to recent research, back-splicing requires both conventional splicing signals and canonical splicing machinery (49, 50).

Two key factors may aid back-splicing circularization: intron pairing and RNA-binding proteins (RBPs). On one hand, intron pairing causes exons to approach one another through ALU elements (51) or nonrepetitive yet complementary sequences (52). Long flanking introns with complementary ALU repeats are essential for developing circHIPK3 (53). On the other hand, some RBPs, such as MBL (MBNL1), QKI, and FUS (54), have the ability to bind firmly and precisely to conserved regions of flanking introns, thus promoting exon circularization and, ultimately, the production of circular RNAs. Furthermore, the action of adenosine deaminase (ADAR) specific to double-stranded RNA (dsRNA) on the editing of adenosine to inosine (50) and the unwinding of the dsRNA helix structure by ATP-dependent RNA helicase A (also known as DHX9) suppresses the synthesis of circRNAs (55, 56). Furthermore, circRNA production may be aided by SFs such as ESRP1 (57) and the elongation velocity of RNA polymerase II (58).

### 4.2 CircRNAs’ Functional Mechanisms

#### 4.2.1 MiRNA Sponge

CircRNAs are involved in a wide range of biological activities because of their distinct structures and other characteristics (59). The capacity of circRNAs to act as miRNA sponges is their most notable function. CircRNAs that include miRNA binding sites bind directly to the matching miRNAs, inhibiting miRNA function and regulating target gene expression. The first circRNA to act as a ceRNA was CDR1as (antisense to the cerebellar degeneration-related protein 1 transcript), which has more than 60 conserved binding sites for miR-7 (44, 60). It bonded to miR-7 and inhibited midbrain growth in zebrafish, comparable to miR-7 knockdown. Hansen et al. hypothesized the same year that mouse circRNA circSRY functioned as a miR-138 sponge with more than ten binding sites (61, 62). Despite the recent identification of numerous circRNAs that operate as miRNA sponges (63, 64), it is essential to note that this represents just a fraction of the hundreds of human circRNAs that have several computational binding sites for specific microRNAs. Interestingly, P. falciparum and S. cerevisiae do not have a miRNA pathway (65), but they do have a large number of circRNAs. As a result, the idea of a miRNA sponge may not represent a universal function for circRNAs.

#### 4.2.2 Splicing or Transcriptional Regulation

Alternative splicing is frequently related to a variety of biological activities (66). CircRNAs were discovered to influence alternative splicing through RNA-mediated interaction, therefore influencing gene expression (67). According to Ashwal-Fluss et al., circMBL, produced from the second exon of the splicing factor muscle blind (MBL/MBNL1), might compete with linear splicing pre-mRNA (50). MBL levels considerably influence circMbl biosynthesis depending on the MBL binding sites. CircMbl has the potential to inhibit the synthesis of its parental mRNA. Another noteworthy example was published in 2015, demonstrating that circRNAs might govern the transcription of the genes they regulate (68). Li et al. discovered that a subclass of circRNA called EIciRNA was mainly located in the nucleus and stimulated transcription of their parental genes by interacting with U1 snRNP, revealing a unique regulatory mechanism for transcriptional control *via* specialized RNA-RNA interactions (68).

#### 4.2.3 Protein Scaffolding and Interaction With RBPs

In addition to MREs, circRNAs include a large number of RBP binding sites. For example, in human cervical cancer HeLa cells, it has been revealed that the antigen R (HuR) interacts with a large number of circRNAs (69). It has been shown that CircPABPN1, which is generated from the PABPN1 gene, may compete with PABPN1 mRNA for binding to HuR, hence limiting the translation of PABPN1. Also discovered were elciRNAs and ciRNAs, which are mainly found in the nucleus and have the ability to bind with small nuclear ribonucleoprotein U1 (snRNP U1) and boost RNA polymerase II (Pol II) transcriptional activity on their parental genes, acting as cis regulators of their respective genes (68, 70). Also of note is that the binding of circRNAs to specific functional proteins may have an impact on several signaling pathways, resulting in alterations in homeostasis (71). In their study, Du et al. discovered that the circular RNA circ-Foxo3 is associated with the anti-senescence protein ID-1, the transcription factor E2F1, and the anti-stress proteins FAK and HIF1α and holds them in the cytoplasm, preventing them from performing their respective duties (72).

#### 4.2.4 Translate Proteins

Although circRNAs are classified as non-coding RNAs, growing evidence suggests that certain circRNAs are translatable (47). Unlike mature mRNA translation, which generally requires the presence of a 5′ end 7-methylguanosine (m7G) cap structure and a 3′ poly(A) tail, circRNA translation is distinct because it does not need the presence of these structures (73). It was proven in 1998 that a circular mRNA containing the whole open reading frame (ORF) of green fluorescent protein (GFP) could effectively encode GFP in Escherichia coli (74). Legnini et al. discovered that circ-ZNF609 includes an ORF and may be translated into a protein in murine myoblasts when activated by IRES (75). Further research found that certain circRNAs containing internal ribosome entry sites (IRESs) may function as the entrance point for ribosomes, therefore triggering translation (76, 77). Surprisingly, the most prevalent RNA modification, N6-methyladenosine (m6A), was discovered to improve circRNA protein-coding capacity (78). According to recent research, FBXW7-185aa, a new 21-kDa protein, was encoded *via* a spanning junction open reading frame in circ-FBXW7 that was directed by an internal ribosome entry site. In glioblastoma, upregulation of FBXW7-185aa suppressed proliferation and cell cycle progression, as well as reduced the half-life of c-Myc by antagonizing USP28-induced c-Myc stabilization, while knockdown of FBXW7-185aa increased malignant phenotypes both *in vitro* and *in vivo*, as previously reported (79). Similarly, Pamudurti N. R. et al. discovered that circMbl could also translate proteins without the need for a cap (80). The protein-coding function sheds new light on the involvement of circRNAs in diseases.

### 4.3 CircRNA: Oncogene or Tumor Suppressor Gene

Emerging evidence suggests that dysregulated circRNAs have both tumor-suppressive and oncogenic roles in cancer origin and development, affecting a wide range of cellular processes. The tumor structure is comprised of cells that have mutations in the genes involved in proliferation and differentiation (81). Oncogenes contribute to the stimulation of cell proliferation. Changes in these genes may vary from the development of novel oncogenes to the overexpression of previously proto-oncogenes. In contrast, tumor suppressor genes (TSGs) inhibit cell growth through the opposite mechanism (82). In this regard, the role of several circRNAs as oncogenes has been investigated. Circ-ABCB10 has been identified as a breast cancer oncogene, and circ-ABCB10 knockdown suppresses proliferation and increases apoptosis in breast cancer cells (83). Similarly, circ-ZEB1 was one of the oncogenic circRNAs that was significantly higher in triple-negative breast cancer (TNBC) tumor tissues and tumor cell lines (84). Circ-ZEB1 knockdown has been shown to reduce TNBC cell proliferation and tumor formation by releasing miR-448 and consequently decreasing the expression of the miR-448 target, eEF2K (84). CircCD44 with oncogenic roles is substantially expressed in TNBC and is adversely correlated with TNBC patient prognosis (85). CircCD44 enhances TNBC proliferation, migration, invasion, and tumorigenesis (85). Remarkably, the oncogenic role of circRNAs in other cancers has also been considered, including circRNA_100859 (86), circAGO2 (87), PIP5K1A (88), circPPP1R12A (89), CCDC66 (90), GLIS2 (91), and circ_0084615 (92) in colon cancer, circRNA-C190 (93), circNDUFB2 (94), circ_100146 (95), circRNA_001846 (96), and circ_0087862 (97) in lung cancer, circAF4 (98), and circSPI1 (99) in leukemia.

On the contrary, studies have shown changes in circRNAs and their functions as TSGs. Circ_0006220 expression was lower in TNBC compared to other subtypes of BC tissues and cell lines. Notably, circ_0006220 functions as a TSG, inhibiting TNBC cell proliferation, migration, and invasion (100). CircCDYL was down-regulated in TNBC tissues, and its expression was found to be positively associated with patient survival rates (101). CircCDYL induces apoptosis and suppresses the proliferation of breast cancer cells with a malignant phenotype (101). The function of circRNAs as TSGs has also been proven in many cancers, including circDDX17 (102), circ_PLXNB1 (103), circTADA2A (104), and Circ-SMARCA5 (105) in colorectal cancer, circ_0004872 (106), circDIDO1 (107), circMAPK1 (108), circ_0035445 (109) and circCCDC9 (110) in gastric cancer, circPLEKHM3 (111) in ovarian cancer, circ-MYBL2 (112) in multiple melanoma, and circSLC8A1 (113) in prostate cancer.

### 4.4 Potentials of CircRNAs in CeRNA Axes

**Figure 3:**
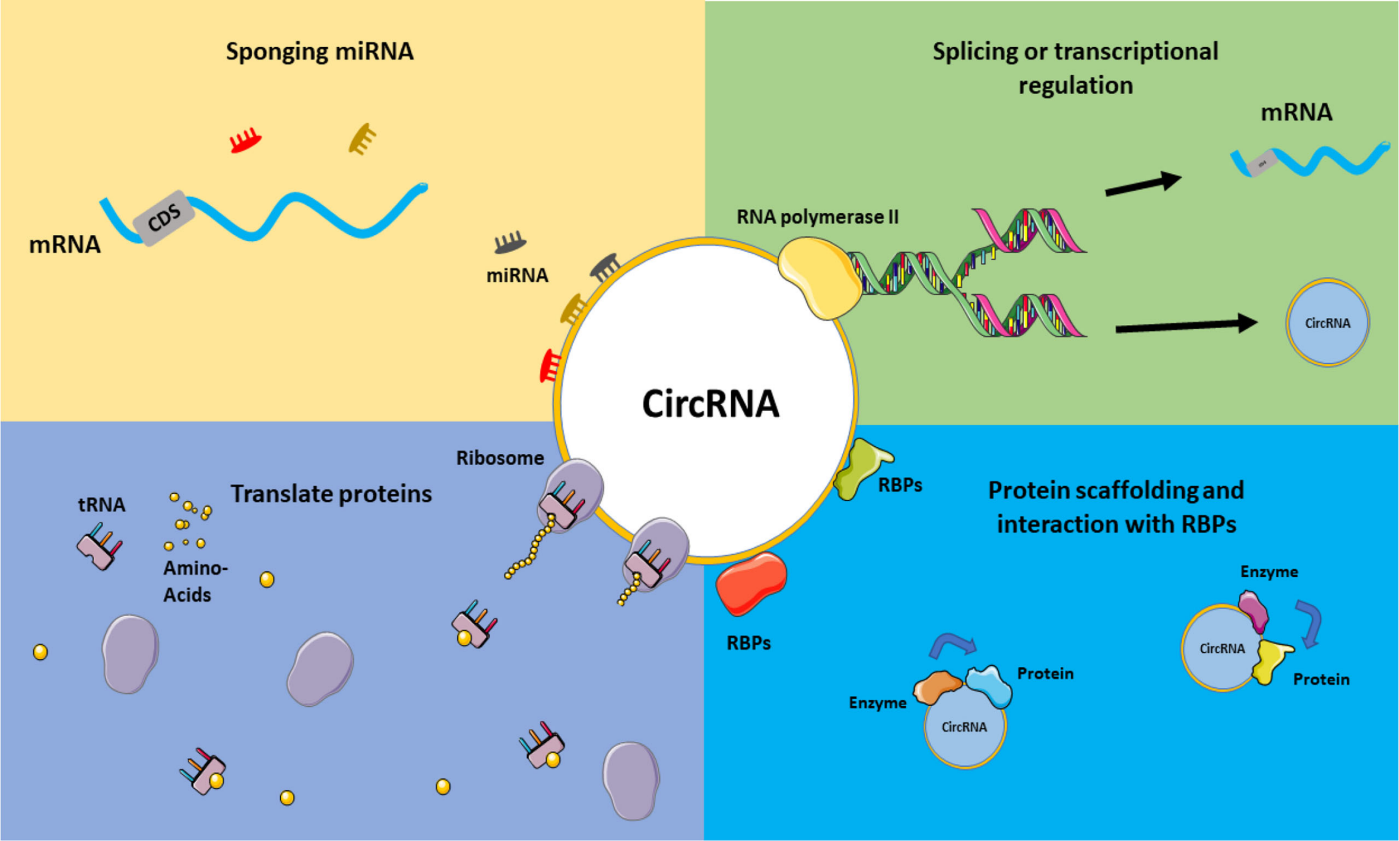
CircRNAs mechanism of actions. Various functions can be considered for circRNAs, including acting in splicing or transcriptional regulations, sponging miRNAs and acting on ceRNA axes, translating proteins and interacting with RBPs, and their role in the protein scaffolding process. This graphical figure was created using the vector image bank of Servier Medical Art (http://smart.servier.com).

Several circRNAs exhibit tissue and developmental stage-specific expression patterns (44). According to recent research, the global abundance of circRNAs was decreased in colorectal cancer (CRC) tissues compared to normal tissues (114). The abundance of a circRNA known as Has-circ-002059 was likewise lower in malignant vs. non-cancerous tissues in gastric cancer (115). Another recent study detected circRNAs in exosomes (small membrane vesicles with endocytic origins released by cells) and discovered that the profiles of seral exosomal circRNAs may identify CRC patients from healthy controls (116). **Figure 3** illustrates a schematic view of the overall function of circRNAs.

The structure of circular RNAs is relatively similar to that of linear RNAs, but they are typically more stable than their linear counterparts because they lack accessible ends and are hence resistant to exonucleases (44). It has been hypothesized that circRNA may create active ceRNA pairs with their linear counterparts due to their unique characteristics like miRNA sponging. CDR1as, a brain-enriched circRNA, has been reported to act as a miRNA sponge for miR-7 in a variety of human disorders (44, 61), including colon cancer (117), gastric cancer (118), esophageal cancer (119), and myocardial infarction (120). Other ceRNA connections discovered in cancer include circPVT1-miR125 in gastric cancer (64), circITCH-miR7/miR214 in lung cancer (121), circHIPK3-miR124 in liver cancer (122), and circTTBK2-miR217 in glioma (123).

**Figure 4:**
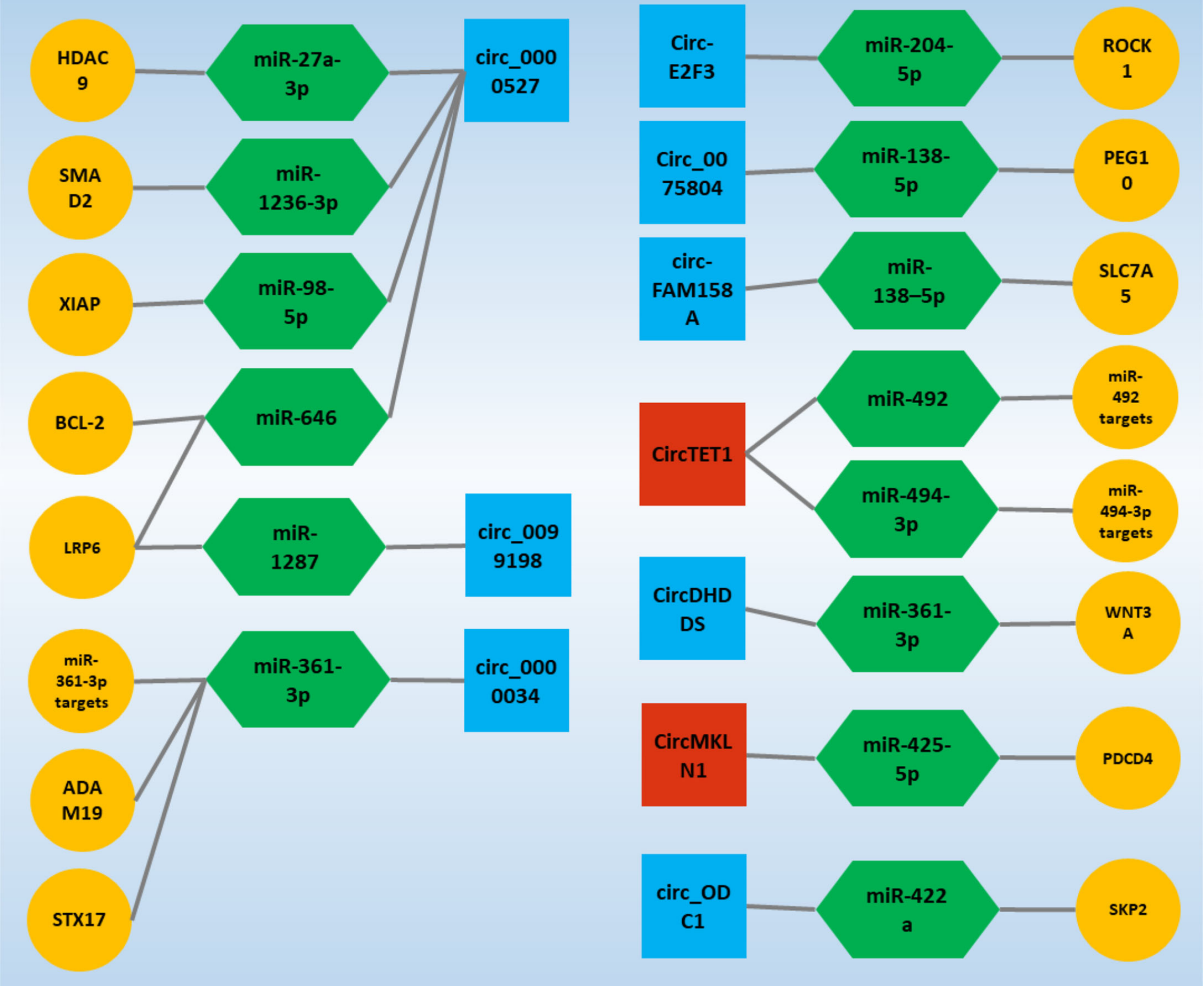
Validated circ-associated ceRNA axes in RB. CircRNAs, miRNAs, and mRNAs are represented by the square, Hexagonal, and circle, respectively. The blue and red colors of the squares indicate an increase and decrease in the expression of circRNAs, respectively. CircRNA, circular RNA; miRNA, microRNA.

In addition, it is interesting that the ceRNA axes in RB have received much attention as a representative of ocular tumors in recent years, and the potential of these regulatory axes is becoming more and more meaningful. On the other hand, studies on axes involving circRNAs in RB are still in their infancy and can play an essential role in the cell by considering the properties of a circRNA. In the following, studies on ceRNA axes in which circRNAs play a crucial role have been obtained in the form of a systematic review study and will be further discussed, and it is expected that this study can be a guide for further studies in this field and cause these studies to receive more attention (**Figure 4**).

### 4.5 Circ_0000527

CircRNA’s function in carcinogenesis and cancer development has received much interest. CircRNA contributes to tumor development by controlling cancer cells’ malignant biological activities such as proliferation, apoptosis, and metastasis (124). Circ_0001649 is claimed to inhibit RB cell proliferation and apoptosis by blocking the AKT/mTOR signaling pathway, and its low expression level implies a bad prognosis in patients (125); circ_ODC1 positively regulates SKP2 and increases RB cell proliferation (25). Surprisingly, it was revealed in another study that the expression level of circ_0000527 in RB tissues and cells was dramatically elevated and that its high expression was highly connected with the degree of differentiation and cTNM staging (30). These findings suggest that circ_0000527 was likely to predict a poor prognosis in RB patients (30). Furthermore, circ_0000527 overexpression significantly increased cell proliferation, motility, and invasion while significantly decreasing cell apoptosis (26). On the other hand, knocking down circ_0000527 had the opposite effect (30, 38). At the same time, miR-646 expression in RB showed a significant decrease, and since miR-646 has several binding sites for circ_0000527, further studies confirmed that circ_0000527 negatively regulates miR-646 expression (26). Interestingly, LRP6, which is regulated by miR-646 and is one of the primary expression genes expressed in malignancies, also experienced a significant increase in expression in the form of a ceRNA axis due to the sponging of miR-646 by circ_0000527 (30). However, studies on increasing the expression of LRP6 in the form of the ceRNA axis in RB were not stopped, and sponge miR-1287 confirmed the increase in the expression of this gene by circ_0099198 in another study (33). Circ_0099198 in RB experiences a significant increase in expression, and this increase in expression shows its effects by decreasing the expression of miR-1287, which is responsible for regulating the expression of LRP6 (33).

Most notably, miR-646 has been identified as a tumor suppressor in a variety of human malignancies. For example, miR-646 inhibits cell growth and migration in lung cancer by negatively regulating the EGFR/Akt pathway (126); in colorectal cancer, miR-646 inhibits cancer development by suppressing NOB1 (127). MiR-646 inhibits the spread of clear cell renal carcinoma cells by downregulating the nin one binding protein (128). MiR-646 suppresses non-small cell lung cancer growth and metastasis through binding to FGF2 and CCND2 (129). In this regard, miR-646 transfection in RB cells was associated with decreased viability, migration, and invasion of these cells (26). Although the oncogenic effects of circ_0000527 have been demonstrated by sponge miR-646, another ceRNA axis was identified by miR-646 targeting BCL-2. Increased circ_0000527 expression by miR-646 sponging increased BCL-2 expression (26), which is one of the significant genes in various malignancies (130).

Circ_0000527 acted as a sponge for miR-1236-3p, which targeted SMAD2 directly, and as a result, miR-1236-3p expression was reduced in RB tissues and cells (37). Circ_0000527 knockdown on RB cell malignancy reduced the effects of MiR-1236-3p inhibition. Furthermore, miR-1236-3p overexpression inhibited RB cell development, whereas SMAD2 elevation reversed the impact (37). Moreover, circ_0000527 knockdown inhibited tumor development *in vivo* (38). Circ_0000527 also sponges miR-98-5p to form another ceRNA axis, promoting the development of RB by increasing the expression of the miR-98-5p target gene, which is XIAP (38).

Nonetheless, investigations in RB on circ_0000527 have demonstrated an increase of this circRNA in the presence of the ceRNA axis, which enhances the expression of genes that lead to the formation and progression of RB and even respond to role treatment sponging particular target miRs. These findings highlight circ_0000527’s significant potential in the processes behind RB and establish circ_0000527 as one of the primary circRNAs in RB.

### 4.6 Circ_0000034

Circ_0000034 is one of the most studied circRNAs in RB. All studies around this circRNA have shown an increase in the expression of this transcript (28, 35, 55). Interestingly, in studies of circ_0000034, the only target of this circRNA was miR−361−3p, whose targets had the potential to influence the mechanisms involved in RB and are essential goals in the process of formation and progression this malignancy. STX17 is a target for miR-361-3p that regulates its expression. Increased circ_0000034 expression due to the binding sites on miR-361-3p sponging miR-361-3p, which increases STX17 expression. On the other hand, the miR-361-3p expression has decreased in studies in RB (27). ADAM19 is another miR-361-3p target gene that experiences increased expression in the circ_0000034/miR-361-3p/ADAM19 ceRNA axis due to miR-361-3p sponging (35). Silencing circ_0000034 also showed that RB progress was suppressed due to circ_0000034’s inability to sponge miR-361-3p. In this condition, miR-361-3p inhibits RB progression by regulating ADAM19 expression (35). Similarly, CircDHDDS, a miR-361-3p molecule sponge, regulated WNT3A expression. As a result, the circDHDDS/miR-361-3p/WNT3A axis promoted RB development by regulating RB cell proliferation, cell cycle program, migration, and invasion (29). In general, since the expression of circ_0000034 increases in RB and because silencing this circRNA inhibits the progression and development of RB, this circRNA can be recognized as one of the main circRNAs in RB.

### 4.7 Other CircRNAs

The ceRNA axes inside the cell are in equilibrium under normal conditions. Increasing or decreasing the expression of each component of an axis can advance the trend in favor of abnormal conditions. The majority of circRNAs studied in the form of ceRNA axes are associated with increased expression, but circTET1 in RB is disturbed by decreased expression of the desired ceRNA axis. It was discovered that CircTET1 expression was downregulated in RB tissues and cells and that its upregulation hampered the growth of RB cells in culture. CircTET1 was shown to be a miR-492/miR-494-3p sponge, and it blocked the Wnt/-catenin pathway *via* miR-492/miR-494-3p (31). Similarly, circMKLN1 expression was reduced in RB tissues and cells. Elevated levels of circMKLN1 were linked to better results in RB patients. CircMKLN1 overexpression inhibited RB cell proliferation, migration, and invasion *in vitro*. MiR-425-5p was shown to be a target of circMKLN1, and it was discovered that increasing miR-425-5p levels might counteract the effects of circMKLN1 overexpression on RB cell malignant tendencies. Furthermore, because circMKLN1 is a target gene for miR-425-5p, suppressing PDCD4 may enhance circMKLN1’s inhibitory effects in RB cell development and metastasis (34).

Nonetheless, the rest of the circRNAs, which have only been studied in one study, have been shown to experience increased expression. Among these, miR-138-5p is considered one of the potential miRs in RB, which is located in specific ceRNA axes due to binding sites for circ_0075804 and circ-FAM158A (36, 39). The circ_0075804/miR-138-5p/PEG10 axis was identified as a ceRNA axis in RB. Through its interaction with miR-138-5p, Circ_0075804 led to the development of the malignant phenotypes of RB cells. Circ_0075804 By sponging, miR-138-5p acts as a barrier to the activity of this miR and cannot regulate the expression of its target gene, which is PEG10, and as a result, PEG10 expression is significantly increased, which acts to cause oncogenic effects in RB (36). Similarly, circ-FAM158A and SLC7A5 were up-regulated, and miR-138-5p was down-regulated in RB. Circ-FAM158A silencing inhibited RB cell proliferation, invasion, and migration while promoting G0/G1 cell cycle arrest and cell apoptosis. Circ-FAM158A functioned as an oncogene in RB by sponging miR-138-5p, which regulates SLC7A5 expression (39).

On the other hand, Circ-E2F3 was shown to be increased in RB tissues and cells, and silencing of circ-E2F3 suppressed RB cell proliferation, migration, invasion, and triggered apoptosis *in vitro*, as well as reduced RB tumor development *in vivo*. Circ-E2F3 sponged MiR-204-5p, and its inhibitor restored the inhibitory impact of circ-E2F3 silencing on RB progression. Furthermore, ROCK1 is known as the target for miR-204-5p, and it is able to control RB development by regulating ROCK1. Moreover, through sponging miR-204-5p, circ-E2F3 favorably regulated ROCK1 expression (32). Finally, given that studies of ceRNA axes centered on circRNAs are at the beginning of the path, and given what genes with potential are involved in these regulatory axes, it will demonstrate the increasing importance of these regulatory axes in RB. The importance of these genes involved in ceRNA axes will be discussed in the following.

### 4.8 Genes Involved in CircRNA-Associated CeRNA Axes

It is desirable to further discuss the genes located in the ceRNA axes in these studies, which are among the genes that have the potential for various cancerous processes. The genes in these ceRNA axes include SMAD2, ROCK1, WNT3A, STX17, LRP6, HDAC9, BCL-2, SLC7A5, ADAM19, XIAP, PEG10, PDCD4, and SKP2. The majority of these genes experience increased expression due to up-regulation of their ceRNA axis circRNA, but PDCD4 experiences reduced expression due to decreased circMKLN1 expression and a reduced ability to sponge miR-425-5p (34). PDCD4 is a new tumor suppressor that exhibits a wide range of actions, including the inhibition of cell proliferation, tumor invasion, metastasis, and the induction of apoptosis. In addition to binding to the translation initiation factor eIF4A and several transcription factors, the PDCD4 protein interacts with a variety of other factors, modulating their functions (131). Decreased expression of this tumor suppressor gene in RB acts in favor of the progression of this malignancy and leaves its effects. Among these, Wnt3A, a member of the Wnt family, is a transcription factor that regulates various cellular processes, including self-renewal, proliferation, differentiation, and motility. Increasing evidence suggests that Wnt3A, depending on the cancer type, either stimulates or inhibits tumor growth *via* the canonical Wnt signaling pathway (132). In RB development, it acts by increasing the expression induced by the circDHDDS/miR-361-3p/WNT3A ceRNA axis by influencing cell proliferation and cell cycle programming, migration, and invasion (29).

Interestingly, SMAD2 is one of the genes that in other cancers, such as prostate cancer (133) and breast cancer (134) are among the genes that appear to play a role in suppressing tumors, and silencing this gene works to promote malignancy (134), it interacts with and promotes RB progression *via* a ceRNA axis, circ_0000527/miR-1236-3p/SMAD2 (37). On the other hand, it is well established that LRP6 regulates breast cancer. LRP6’s expression is most often up-regulated in breast cancer tissue, and overexpression or knockdown of LRP6 causes or halts breast tumorigenesis (135). In line with the study conducted in RB, the expression of LRP6 in the form of a ceRNA axis also experiences an increase in expression, and in the direction of RB development, this increase in expression demonstrates its oncogenic effects (30, 33). SKP2 (S-phase kinase-associated protein 2) is an SCF E3 ligase-specific factor promoting cell cycle progression by degrading its targets. SKP2 substrates other than p27 have been found, including p57 (136), p130 (137), p21 (138), Tob1 (139), and FOXO1 (140). These substrates have a role in various biological activities, including cell cycle regulation, growth, differentiating, apoptosis, and survival. SKP2 has an undeniably essential role in controlling various cellular processes owing to the degradation of its substrates, the majority of which are tumor suppressor proteins (141). SKP2 is hypothesized to operate as an oncoprotein since it is responsible for the degradation of the tumor, as mentioned earlier, suppressor proteins. SKP2 overexpression has been found in a wide range of human malignancies, including lymphomas (142), prostate cancer (143), colorectal cancer (144), melanoma (145), nasopharyngeal carcinoma (146), pancreatic cancer (147), and breast carcinomas (148). In addition, the expression of SKP2 in RB in the form of a ceRNA axis also experiences an increase in expression due to the effect of circ_ODC1 on miR‐422a and the oncogenic effects of SKP2 in RB appear (25).

It was recently discovered that ROCK1 plays a critical role in regulating cell motility, angiogenesis, and migration (149). Furthermore, ROCK1 has been found to be overexpressed in a variety of solid tumors, including glioblastoma (150), melanoma (151), osteosarcoma (152), and hepatocellular carcinoma (153). According to published reports, ROCK1 overexpression is strongly associated with more highly metastatic and invasive phenotypes in human cancers (149). In this regard, ROCK1 is overexpressed in RB as part of a ceRNA axis, which promotes RB proliferation, metastasis, and invasion while decreasing apoptosis (32). Similarly, according to several research findings, HDAC9 has been implicated in the development of malignant tumors. HDAC9 overexpression has been linked to cell growth in medulloblastoma (154), lymphoma (155), oral squamous cell carcinoma (156), and breast cancer (157). It should be noted that the overexpression of this gene in RB was also proven in a study, but this increase in expression was not detected in the form of a ceRNA axis (158). HDAC9 experiences increased expression in the form of the circ_0000527/miR-27a-3p/HDAC9 ceRNA axis and exerts its oncogenic properties by activating the PI3K/AKT pathway (40). STX17 (Syntaxin 17) is an autophagy-related ER-resident SNARE (soluble N-ethylmaleimide-sensitive factor attachment protein receptor) protein. Various roles for STX17 in terms of its mechanisms of action have been proposed (159).

XIAP overexpression in tumor cells has been linked to tumor aggressiveness and has been described as a mediator of resistance to chemotherapy and targeted therapy in various malignancies (160). In RB, the increase in XIAP expression also occurs due to the sponging of miR-98-5p by circ_0000527, which acts in the direction of the oncogenic properties of XIAP (38). Likewise, PEG10 appears to play a critical role in tumor growth in a variety of cancers, including hepatocellular carcinoma (161), lung cancer (162), and prostate cancer (163). In RB, miR-138-5p is sponged by circ_0075804 and forms a ceRNA axis with one of the targets of miR-138-5p, PEG10, which increases its expression and leads to a malignant phenotype (36). Bcl-2-family proteins play critical roles in cell death regulation, regulating various cell death mechanisms such as apoptosis, necrosis, and autophagy (164, 165). Increased viability, metastasis, and invasion in RB occur due to increased expression levels of BCL-2 in the form of the ceRNA axis circ_0000527/miR-646/BCL-2 (26). SLC7A5 imports essential amino acids into cancer cells, and research has shown that amino acids, particularly leucine, activate mTORC1, which regulates protein translation and cell growth, and inhibits apoptosis in malignant cells by activating mTORC1 (166, 167). Circ-FAM158A and SLC7A5 are overexpressed in RB. SLC7A5 acts as a lever to demonstrate the oncogenic properties of circ-FAM158A (39).

As mentioned, the genes considered so far in the studies, including genes with potential and influence in the processes involved in cancer, play an essential role in the development of RB. The localization of these essential genes in the ceRNA axes indicates the potential of these axes in cancer causation and emphasizes the principle that molecular mechanisms, especially ceRNA axes, should be further studied.

## 5 Limitations

The present study had several limitations. On the one hand, ceRNA studies centered on circRNAs are just beginning, and the number of such studies is limited. This study tried to be a roadmap for further studies in this field and increase attention and enthusiasm for future studies in RB. On the other hand, we attempted to cover all of the details pertaining to these regulatory axes and circRNAs in **Table 1** in the section on key findings. All care was taken not to miss a study during the screening process, and three persons collaborated on this part so that this study would include all of the studies conducted in the field of RB, but this is conceivable due to an individual error that may have left a study off the table.

## 6 Conclusion

Numerous circRNAs have been discovered and studied in recent years. CircRNAs act as miRNA sponges, influencing the expression of related genes and forming a regulatory axis called ceRNA. CeRNA axes are potential axes that function from disease onset to diagnosis and treatment. This study gathered evidence that circRNAs had a fascinating impact on essential genes in RB, each of which has the potency to be a defining characteristic of this cancer. Thus, our efforts to understand different aspects of ceRNA regulation processes in RB pathogenesis provide new insights into potential molecular targets, identify ceRNA-based diagnostics, and develop ceRNA-based therapeutic potential.

## Author Contributions

MT and MA wrote the draft and revised it. MR and AR designed and supervised the study. BH, HS, MM, MS-B and PH collected the data and designed the figures and tables. All the authors read and approved the submitted version.

## Conflict of Interest

The authors declare that the research was conducted in the absence of any commercial or financial relationships that could be construed as a potential conflict of interest.

## Publisher’s Note

All claims expressed in this article are solely those of the authors and do not necessarily represent those of their affiliated organizations, or those of the publisher, the editors and the reviewers. Any product that may be evaluated in this article, or claim that may be made by its manufacturer, is not guaranteed or endorsed by the publisher.

## Abbreviations

RB, Retinoblastoma; ceRNA, Competing endogenous RNA; CircRNA, Circular RNA; MRE, MicroRNA Response Element; PRISMA-ScR, Preferred Reporting Items for Systematic Reviews and Meta-Analyses Extension for Scoping Reviews; RBP, RNA-binding protein; dsRNA, double-stranded RNA; snRNP, small nuclear ribonucleoprotein; ORF, open reading frame; GFP, green fluorescent protein; CRC, Colorectal Cancer; TSGs, tumor suppressor genes; TNBC, triple negative breast cancer.
